# Direct Prediction of 48 Month Survival Status in Patients with Uveal Melanoma Using Deep Learning and Digital Cytopathology Images

**DOI:** 10.3390/cancers17020230

**Published:** 2025-01-13

**Authors:** T. Y. Alvin Liu, Haomin Chen, Neslihan Dilruba Koseoglu, Anna Kolchinski, Mathias Unberath, Zelia M. Correa

**Affiliations:** 1 Wilmer Eye Institute, School of Medicine, Johns Hopkins University, Baltimore, MD 21287, USA; 2 School of Engineering, Johns Hopkins University, Baltimore, MD 21218, USA; 3 School of Medicine, Johns Hopkins University, Baltimore, MD 21287, USA; 4 Ocular Oncology Service, Bascom Palmer Eye Institute, University of Miami Miller School of Medicine, Miami, FL 33136, USA

**Keywords:** uveal melanoma, patient survival, prediction, deep learning, artificial intelligence, cytopathology

## Abstract

Background: Uveal melanoma (UM) is the most common primary intraocular malignancy in adults. The median overall survival time for patients who develop metastasis is approximately one year. In this study, we aim to leverage deep learning (DL) techniques to analyze digital cytopathology images and directly predict the 48 month survival status on a patient level. Methods: Fine-needle aspiration biopsy (FNAB) of the tumor was performed in each patient diagnosed with UM. The cell aspirate was smeared on a glass slide and stained with H&E. Each slide then underwent whole-slide scanning. Within each whole-slide image, regions of interest (ROIs) with UM cells were automatically extracted. Each ROI was converted into super pixels, and the super pixels were automatically detected, segmented and annotated as “tumor cell” or “background” using DL. Cell-level features were extracted from the segmented tumor cells. The cell-level features were aggregated into slide-level features which were learned by a fully connected layer in an artificial neural network, and the patient survival status was predicted directly from the slide-level features. The data were partitioned at the patient level (78% training and 22% testing). Our DL model was trained to perform the binary prediction of yes-versus-no survival by Month 48. The ground truth for patient survival was established via a retrospective chart review. Results: A total of 74 patients were included in this study (43% female; mean age at the time of diagnosis: 61.8 ± 11.6 years), and 207,260 unique ROIs were generated for model training and testing. By Month 48 after diagnosis, 18 patients (24%) died from UM metastasis. Our hold-out test set contained 16 patients, where 6 patients had passed away and 10 patients were alive at Month 48. When using a sensitivity threshold of 80% in predicting UM-specific death by Month 48, our model achieved an overall accuracy of 75%. Within the subgroup of patients who died by Month 48, our model achieved a prediction accuracy of 83%. Of note, one patient in our test set was a clinical surprise, namely death by Month 48 despite having a GEP class 1A tumor, which typically portends a good prognosis. Our model correctly predicted this clinical surprise as well. Conclusions: Our DL model was able to predict the Month 48 survival status directly from digital cytopathology images obtained from FNABs of UM tumors with reasonably robust performance. This approach, if validated prospectively, could serve as an alternative survival prediction tool for patients with UM to whom GEP is not available.

## Simple Summary

Uveal melanoma (UM) is the most common primary intraocular malignancy in adults. Local treatment of tumors is typically straightforward. Yet, despite successful local treatment, a portion of patients will still develop subsequent distant metastases which are usually fatal. The current gold standard tool for prognostication is gene expression profiling (GEP), which is only available in the United States. The goal of this research is to develop an artificial intelligence (deep learning-based) tool which can predict patient survival directly from digital cytopathology images generated from fine-needle aspiration biopsies. Our deep learning model was able to predict Month 48 survival status directly from digital cytopathology images with reasonably robust performance. This approach, if validated prospectively, could serve as an alternative survival prediction tool for patients with UM to whom GEP is not available.

## 1. Introduction

Uveal melanoma (UM) is the most prevalent primary intraocular tumor among adults [1]. According to the Surveillance, Epidemiology, and End Results (SEER) Program of the National Cancer Institute (NCI), the 5 year relative survival rates for patients with UM are significantly influenced by metastasis. While the overall 5 year relative survival rate is estimated to be 79% (95% CI: 73–88%), the median overall survival time for patients who develop metastasis is much shorter at approximately 1.07 years (range: 0.59–2.50 years), even after any kind of treatment [2,3]. Systemic metastases are present in only 2–3% of patients at the initial time of diagnosis, and these patients have a poor survival rate at 5 years (16%) [4,5].

Since tumor genetics drives metastasis and affects patient survival in UM, much research on patient risk stratification using genetic analyses has been performed. The Cancer Genome Atlas (TCGA) categorized UM into four prognostic groups based on DNA alterations alone: Group A (chromosome 3 disomy and chromosome 8 disomy), Group B (chromosome 3 disomy and chromosome 8q gain), Group C (chromosome 3 monosomy), and Group D (chromosome 3 monosomy and multiple chromosome 8q gains) [6,7]. In a validation study, the 5 year rate of metastasis progressively increased from 4% for Group A to 20% for Group B, 33% for Group C, and 63% for Group D (*p* < 0.001) [8]. In addition, mutations in specific genes, such as BAP1, GNAQ, and GNA11, have been shown to be associated with more aggressive disease and poorer survival outcomes. For example, Guanosine Nucleotide-Binding Protein Q gene (GNAQ) and its paralogue, Guanosine Nucleotide-Binding Protein Alpha-11 Gene (GNA11), are mutually exclusive oncogenic genes which are present in 80-90% of UM patients. Griewank et al. conducted a study demonstrating that GNAQ mutations were identified in patients with UM metastases of the liver, lymph nodes, and skin. Tumors with GNA11 mutations were associated with poorer disease-specific and overall survival rates compared with tumors without the mutation [9,10]. In recent years, gene expression profiling (GEP) has emerged as the most robust test for predicting long-term survival rates in these patients, independent of other clinicopathological parameters. GEP can be assessed for samples obtained through fine-needle aspiration biopsy (FNAB) of the tumors and can be broadly classified into two groups. While patients with GEP class 1 tumors show a 92-month survival probability of 95%, patients with GEP class 2 tumors have a significantly lower probability of 31% [11]. GEP class 1 tumors can be further divided into 1A and 1B, with a better prognosis associated with class 1A tumors.

However, the aforementioned genetic analyses are not readily available everywhere in the world. Given the limited availability of these genetic tests, we previously investigated whether it was possible to use deep learning (DL) techniques to predict the genetic information of a particular UM tumor. DL is a subset of machine learning, which is a type of artificial intelligence (AI). DL is currently the gold-standard AI technique for medical image analyses, is highly effective in the extraction of complex image features, and has been applied in image classification, object detection, and segmentation [12,13]. Our group previously demonstrated the feasibility of using DL techniques to directly predict the GEP status from digital cytopathology images obtained from FNABs, and our best-performing model achieved an accuracy of 91.7% on a patient level [11,14]. As a follow-up to our previous work, the objective of this study is to directly predict the 48-month survival status in patients with UM from digital cytopathology images.

## 2. Methods

We conducted a retrospective study involving deidentified digital cytopathology whole-slide images from 74 consecutive patients with UM. The data were split randomly on the patient level, with 58 patients (78%) for model training and 16 patients (22%) for model testing. This study adheres to the Declaration of Helsinki, and it was approved by the Johns Hopkins University Institutional Review Board as an exempt-from-consent study because the slides and patient information were deidentified. The clinical ground truth for survival status at Month 48 after initial diagnosis was established via a chart review of the electronic health records.

### 2.1. Region of Image (ROI) Preparation

Each of the 74 patients underwent plaque brachytherapy for local treatment of the UM tumor. Prior to the placement of the plaque in the operation room, two FNABs of the tumor were performed. The first biopsy was taken at the tumor apex for GEP (DecisionDx-UM^®^, Castle Biosciences, Friendswood, TX, USA), and the second biopsy was taken near the tumor apex for cytopathology slide generation. The cytology specimen was flushed on a standard pathology glass slide, smeared, and stained with hematoxylin and eosin (H&E). Whole-slide scanning was performed for each cytology slide at a magnification of 40×.

Next, we applied a human-interactive, computationally assisted tool to automatically extract high-quality regions of interest (ROIs) from each whole-slide image [15]. This computationally assisted tool was established using a deep clustering algorithm. After blank image regions were eliminated, all other image regions were divided into 100 cluster centroids. Image regions within each cluster centroid were annotated by human pathologists as “high”, “low”, or “mixed” quality. These annotations were then used to train the deep clustering algorithm. The size of each ROI was 256 × 256 pixels, and in total, 207,260 unique ROIs were extracted from all slides.

### 2.2. Predictive Model Development

**Figure 1:**
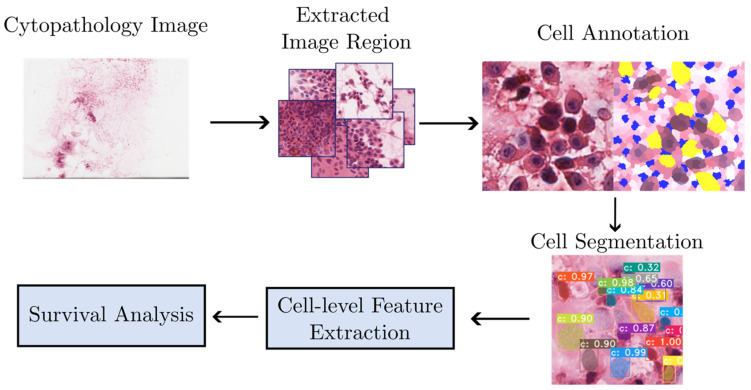
Overall model development pipeline.

The entire model development pipeline is shown in Figure 1.

Of the 207,260 ROIs, 500 were randomly selected from 85 whole-slide images, and within each of these 500 ROIs, super pixels [16] were generated and annotated as either “tumor cells” or “background”. Each UM tumor cell typically comprised several super pixels, and the super pixel annotations within each cancer cell were grouped together to form instance-level annotations, which were then used to train YOLACT [17] for instance-level segmentation and object detection. The YOLACT model is an end-to-end instance segmentation model. First, it generates heatmaps as prototypes. Then, these prototypes are linearly combined with each other to create new heatmaps for each detected object. Finally, the new heatmaps are thresholded to generate detection segmentation masks. The performance of the YOLACT segmentation model was evaluated using the mean average precision (mAP) metric, which was dependent on the intersection over union (IoU) threshold. Our YOLACT model achieved an mAP of 70.7%, with an IoU of 0.5. The fully trained instance-level segmentation network was used to process all the ROIs. For each detected and segmented tumor cell, we extracted the cell-level feature from the last convolutional layer by using the segmentation mask $M_{c}$ and applying mask average pooling for the encoded image feature F:

$$F_{c}=\mathrm{Avg}\left(F\left[M_{c}\right]\right)$$

Here, $F_{c}$ is the cell-level feature of cell c.

The cell-level features were aggregated into slide-level features, and our artificial neural network (ANN) was trained to predict the patient survival status directly from the slide-level features.

**Figure 2:**
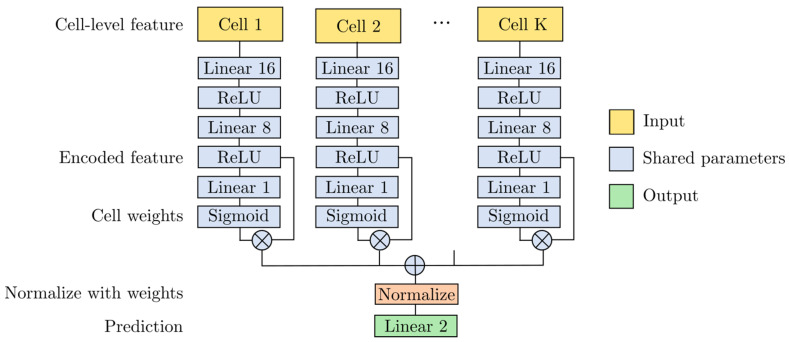
ANN used for aggregating cell-level features and predicting survival status from slide-level features.

A schematic representation of the ANN is shown in Figure 2.

We encoded each cell-level feature into a length-8 feature. The encoded cell-level features were then averaged by the cell weights to form the slide-level features, which were normalized by a sigmoid function in a fully connected layer. The cell weights were not predefined to allow the network to learn in an unbiased fashion. The cell weights were generated by an attention mechanism in the ANN, using the cell’s own DL feature. Finally, the slide-level feature was learned to generate survival predictions.

## 3. Results

In total, 74 patients and 207,260 unique ROIs (image tiles generated from cytopathology digital whole-slide images) were included in our study. Among the patients, 43% (32/74) were female, and the mean age at the time of diagnosis was 61.8 ± 11.6 years. All patients received plaque brachytherapy for local treatment of their UM tumors. Of the 74 tumors, 28% belonged to GEP class 1A (21/74), 20% belonged to GEP class 1B (15/74), and 52% belonged to GEP class 2 (38/74). Overall, 30% (22/74) of the tumors had ciliary body involvement. The mean largest basal diameter and thickness of the tumor were 11.8 (±3.9) mm and 5.9 (±3.2) mm, respectively (Table 1).

By Month 48 after initial diagnosis, 24% (18/74) of the patients had died from UM metastasis. Of the patients who were still alive at Month 48, the mean follow-up duration was 5.5 ± 0.9 years. Our hold-out test set contained 16 patients. Of these 16 patients, 6 had passed away by Month 48 (one with a class 1A tumor and five with class 2 tumors). Of these 16 patients, 10 were still alive at Month 48 (3 with class 1A tumors, 3 with class 1B tumors, and 4 with class 2 tumors).

During internal validation using the training dataset only, our model achieved an AUC of 0.95. During model testing, when using the hold-out test set and a sensitivity threshold of 80% in predicting UM-specific death by Month 48, our model achieved an overall accuracy of 75%, a 71.4% F1 score, 83.3% recall, and 62.5% precision on a patient level. Within the subgroup of patients who died by Month 48 from UM metastasis, our model achieved a prediction accuracy of 83%. Of note, one patient in our test set was a clinical surprise. This patient died by Month 48 despite having a GEP class 1A tumor, which typically portends a good prognosis. Our model correctly predicted this clinical surprise as well.

## 4. Discussion

In this study, we developed an alternative survival prediction tool for patients with UM based on DL techniques and digital cytopathology images. The current gold standard for UM prognostication is GEP. Similar to GEP, the pipeline we developed involves performing FNAB of the tumor. However, in contrast to GEP, our tool may offer several advantages. First, GEP (DecisionDx-UM^®^) is only available in the United States, whereas our model could be housed in the cloud and be accessed anywhere in the world with an internet connection. Second, GEP typically costs thousands of dollars and has a turnover time of days, whereas generating a prediction with our DL model only takes minutes and has a low marginal cost (the cost of running the model on a computer server). Third, while GEP can provide an estimated chance of survival based on extrapolation from cohort-level data [18], our model provides a more personalized prediction on a patient level and a more specific prediction with a binary outcome (survival: yes versus no). Taken together, our data suggest that our model, if validated prospectively, could serve as a complementary or alternative tool to GEP, the current gold standard prognostication test for UM.

In the past few years, there has been increasing interest in using DL techniques for detection, classification, and prognostic prediction of UM. For example, DL has been used to distinguish benign melanocytic lesions from UM in color fundus photographs [19,20]. One of the most promising DL applications in UM involved the analysis of digital pathology images [19,20]. Our group was among the first to explore the use of DL to predict genetic information in UM tumors. Specifically, we demonstrated that the GEP status could be directly predicted from digital cytopathology images [11,14]. In addition to GEP, mutation in the BAP1 gene has also been a focus, as mutations in the BAP1 gene are frequently encountered in metastasizing UM [21]. For example, Sun et al. and Zhang et al. demonstrated that DL could effectively predict BAP1 gene expression in digital histopathology images stained with H&E [22,23]. Similarly, Akram et al. studied the efficacy of using a DL model to predict molecular subclasses (BAP1, SF3B1, and EIF1AX), in comparison with gold-standard molecular testing, and they concluded that DL was able to identify distinct histopathological features for each subclass, such as an association between epithelioid cellular morphology and BAP1 [24]. However, in contrast to our studies, which analyzed cytopathology images, the studies by Sun, Zhang, and Akram et al. analyzed histopathology images of UM tumors, thus limiting their practicality. Cytopathology images can be obtained using FNABs, which are frequently performed to confirm a diagnosis or provide cellular aspirates for prognostication purposes, whereas obtaining histopathology images of UM tumors requires enucleation of the globe, which is rarely performed in present day management of UM.

DL has been used to analyze digital pathology images in other malignancies as well. For example, DL has been used to predict patient survival from digital pathology images in gastric cancer [25], colorectal cancer [26], and small-cell lung cancer [27]. Within the context of ophthalmology and UM, Wan et al. classified UM patients into two clusters based on distinct survival outcomes by using 14 histopathological features extracted via DL [28]. While this approach of using DL to extract specific histopathological features provided biomarker information and improved model explainability, it is still limited by the need for histopathologic image generation and can only provide survival probability on a cohort level. In contrast, our current study, to our knowledge, is the first to be able to provide survival prediction on a patient level using digital cytopathology images.

In addition, in our technical pipeline, the UM cells in the ROIs were first detected and segmented, and the extracted cell-level features were further aggregated and used for survival prediction. We designed this pipeline to ensure that the final survival prediction would be influenced by UM cell morphology, and our approach was inspired by the well-known fact that UM cell morphology is correlated with prognoses. For example, tumors with epithelioid cells portend worse prognoses compared with tumors with spindle cells. One particularly encouraging aspect of our results was our model’s ability to correctly predict the unexpected early death (2.3 years after diagnosis) of a patient with a class 1A tumor per GEP. Class 1A tumors carry the best prognosis, and the predicted survival probability is reported to be 98% at 3 years [18]. Our model’s ability to directly predict survival prognoses has important clinical implications. Patients with UM typically expire from distant UM metastasis months or years after the initial successful local treatment of a tumor. Once patients with a high chance of non-survival (i.e., high chance of future metastasis) are identified, the management plan can then be individualized. For example, patients at a high risk of future metastasis could be monitored more frequently with MRI images of the liver, the most common site for UM metastases which are typically fatal.

Our study has several limitations. First, although FNABs of UM tumors are quite routinely performed in academic centers in the United States, it is still a specialized skill set which some ocular oncologists may not be as familiar with. Second, our sample size was relatively small, and our data were collected retrospectively. Our model will require further validation with a prospective external patient cohort. Third, the patients in our cohort who expired from metastatic disease by Month 48 of diagnosis all died prior to the FDA approval of Tebentafusp (Kimmtrak^®^). Tebentafusp is an immunotherapy drug approved in 2022 for treatment of metastatic UM. Most prognostication tools, including GEP and our DL model, will likely benefit from updates and fine-tuning with new data with the advent of new classes of therapy such as Tebentafusp.

## 5. Conclusions

In conclusion, we developed a DL-based, end-to-end pipeline which was able to predict the 48 Month survival status directly from digital cytopathology images obtained from FNABs of UM tumors with reasonably robust performance. There is preliminary evidence to suggest that our approach could predict adverse clinical surprises, namely early death in patients who are supposed to perform well through GEP. Our model, if validated prospectively with a larger, more diverse patient cohort, could serve as an alternative or complementary survival prediction test for patients with UM.

## Figures and Tables

**Table 1:** Baseline demographic and clinical characteristics of the 74 patients included in the current study.

% Female	Mean Age at Diagnosis	% GEP Class 1A	% GEP Class 1B	% GEP Class 2	% Ciliary Body Involvement	Mean Tumor Largest Basal Diameter	Mean Tumor Thickness	% Survival at Month 48
43	61.8 ± 11.6 years	28	20	52	30	11.8 ± 3.9 mm	5.9 ± 3.2 mm	76

## Data Availability

Code and data inquiries may be submitted to the corresponding authors and will be reviewed on a case-by-case basis.
